# Biologically Synthesized Gold Nanoparticles with Enhanced Antioxidant and Catalytic Properties

**DOI:** 10.3390/ph17091105

**Published:** 2024-08-23

**Authors:** Melinda David, Teodor A. Enache, Lucian Barbu-Tudoran, Camelia Bala, Monica Florescu

**Affiliations:** 1 Laboratory for Quality Control and Process Monitoring, University of Bucharest, 4-12 Elisabeta Blvd., 030018 Bucharest, Romania; melinda.david@s.unibuc.ro; 2 Department of Fundamental, Prophylactic and Clinical Disciplines, Faculty of Medicine, Transilvania University of Brasov, Str. Universitatii no. 1, 500068 Brasov, Romania; 3 National Institute of Material Physics, Atomistilor 405A, 077125 Magurele, Romania; adrian.enache@infim.ro; 4 Electron Microscopy Center “C. Craciun”, Biology and Geology Faculty, Babes-Bolyai University Cluj-Napoca, 4-6 Clinicilor Str., 400006 Cluj-Napoca, Romania; lucian.barbu@ubbcluj.ro; 5 National Institute for R&D of Isotopic and Molecular Technologies (INCDTIM) Cluj-Napoca, 67-103 Donath Str., 400293 Cluj-Napoca, Romania; 6 Department of Analytical Chemistry and Physical Chemistry, University of Bucharest, 4-12 Elisabeta Blvd., 030018 Bucharest, Romania

**Keywords:** biological synthesis, metallic nanoparticles, enhanced antioxidant properties, enhanced catalytic properties

## Abstract

Increasing levels of reactive oxygen species generate oxidative stress in the human body that can lead to various medical conditions. The use of nanomaterials exhibiting antioxidant properties may prevent these effects. The biological synthesis of metallic nanoparticles using plant extracts with antioxidant properties can offer benefits due to their active compounds. The used extracts contained reducing and stabilizing agents, which were shown to be transferred onto the gold nanoparticles, functionalizing them. Herin, we report a gold nanoparticle synthesis by eco-friendly biological methods (b-AuNPs) using extracts of sea buckthorn, lavender, walnuts, and grapes, obtained through ultrasound-assisted extraction and pressure-enhanced extraction. The obtained b-AuNPs were characterized by UV–Vis and FTIR spectroscopies and visualized using transmission electron microscopy. The catalytic and scavenging effect of the b-AuNPs towards H_2_O_2_ (as reactive oxygen species) was evaluated electrochemically, highlighting the protective behavior of b-AuNPs towards lipid peroxidation. All experiments demonstrated the stability and reproducibility of prepared b-AuNPs with enhanced antioxidant and catalytic properties, opening a new perspective for their use in biomedical applications.

## 1. Introduction

Natural antioxidant compounds are abundant in the surrounding environment, providing a sustainable route for the synthesis of nanomaterials. The green synthesis of nanomaterials can offer various benefits, such as the active compounds from plants, microbes, or fungi, which contain reducing and stabilizing agents, tailoring the functionality of nanoparticles (NPs), reduced energy input, and lower production costs [1]. With nanotechnology gaining more interest in medical applications, biological routes for NP synthesis enhance their antioxidant, antibacterial, or catalytic properties [2]. However, the potential of plants for NP synthesis has not yet been fully explored due to the vast diversity and composition of plants. Although plants known to incorporate antioxidant compounds are targeted for the biological synthesis of NPs, their use in actual biomedical applications is still limited [3].

Extracellular synthesis is a simple procedure that accomplishes the plant-mediated synthesis of metallic NPs, where an aqueous plant extract is mixed with an aqueous metal salt solution. In this case, the reduction of the metal ions occurs slowly, in time [4]. Metal oxides and metal NPs obtained following this approach showed spectacular advantages in size, shape, composition, and physico-chemical properties [5]. The most investigated metals in this green synthesis are silver (Ag) [6], gold (Au) [7,8], and zinc oxide (ZnO) [9]. The reduction of the metal oxide and metal salts is produced by various biomolecules in the plant extracts, such as proteins, sugars, enzymes, amino acids, and other metal traces [10]. Plant extracts are commercially available or can be easily obtained through drying, boiling, and filtering, or by ultra-sound-assisted extraction or rapid pressurized extraction from different plants as whole or parts [11,12]. The shape and size of biosynthesized NPs strongly depend on the nature of the biological compounds and their content in the plant extracts. Moreover, the NP growth medium parameters such as pH, temperature, exposure time, and salt concentration also play an important role [13].

The research has mainly focused on the biological synthesis of gold nanoparticles (b-AuNPs) and their characterization, while real-life applications have been addressed less. This is mainly caused by many unknown variables during biological synthesis. For AuNPs, it was shown that these can both promote or repress cell viability, depending on the physical parameters [14]. In addition, AuNPs have been shown to possess excellent enzyme-like activity [15], providing many active sites, and hence can be used in cosmetics and wound healing by enhancing cell viability. On the other hand, higher concentrations of AuNPs or AuNPs incorporated in nanostructured hybrid materials could be applied to selectively kill cancer cells [16]. Thus, a remaining challenge is the tuning of AuNPs in such a manner as to lower their high surface energy, increase stability, and prevent aggregation [17].

Our research aims to explore biological methods for synthesizing AuNPs to address various challenges. The primary uses for biologically synthesized AuNPs include therapeutics [18], drug delivery [19], and sensing [20]. This study aims to compare AuNPs synthesized using eco-friendly methods with aqueous plant extracts from various plants. We used two extraction methods to obtain the plant extracts: ultrasound-assisted extraction (US) and pressure-enhanced extraction (T). Sea buckthorn (Hf), lavender (Lf), walnuts (Js), and grapes (Vp) are all plants with antioxidant properties, and we believe that compounds in these plants can be used to reduce gold metal salt into biologically synthesized AuNPs. There are several studies showing that all parts of sea buckthorn (fruit, leaves, bark, and seeds) have great medicinal and therapeutic potential. It is also widely used in the human food industry since it is a promising economic plant [21]. Lavender is a strongly aromatic shrub shown to have mainly therapeutical properties, such as neuroprotective effects, antiseptic, anti-inflammatory, and analgesic properties [22]. Similarly, grape seed and skin extracts have therapeutic properties and pro-proliferative effects. It has been shown that they reduce the level of oxidative stress and improve the overall lipid metabolism [23]. Walnuts also contain several anti-inflammatory and antioxidant components; they can reduce oxidative stress and oxidative damage to lipids [24]. All of these plants are associated with (poly)phenolic substances with high biological activity. It has been demonstrated that these compounds can reduce metal ions and promote the development of AuNPs. Furthermore, due to their electrocatalytic properties, biologically synthesized AuNPs may have diverse biomedical applications: antioxidant, antibacterial, and anticancer characteristics for therapeutic applications (photothermal therapy and targeted drug delivery) and biosensing applications. B-AuNPs also enable functionalization with specific biomolecules, and when incorporated into tissue engineering scaffolds or hydrogels, they can change cellular behavior by encouraging cell adhesion, proliferation, and differentiation [25]. Thus, functional groups (-OH) transferred on the AuNP surface from the used plant extracts have antioxidant properties, which is highlighted through their capability to scavenge reactive oxygen species (ROS) such as hydrogen peroxide (H_2_O_2_). Free radicals, e.g., ROS, are molecules containing oxygen and one or more unpaired electrons, which makes them very reactive. In organisms, radicals are generated in metabolic and physiological processes. Some of the most important ROS are the radicals of hydroxyl, superoxide anion, peroxynitrite or hydrogen peroxide (H_2_O_2_), and singlet oxygen. The imbalance between ROS and antioxidant molecules (AOx) leads to oxidative stress, which has been correlated to various disorders caused by damage to healthy cells, DNA, and protein molecules and which further initiate lipid peroxidation. Thus, biologically synthesized AuNPs bearing antioxidant molecules possess the capability to scavenge the overproduction of ROS [26,27].

AuNPs are widely used for the detection of H_2_O_2_ [28]. However, biologically synthesized AuNPs are best suited for biomedical applications due to their increased catalytic properties and antioxidant capacity. To explore the catalytic properties of b-AuNPs and their application, screen-printed carbon electrodes were modified with colloidal solutions of b-AuNPs by drop-casting. Using liposomal systems as simulated cell membranes in combination with electrochemical impedance spectroscopy (EIS), the beneficial effect of b-AuNPs was highlighted upon H_2_O_2_ addition. Further, the antioxidant activity of the b-AuNPs was validated in vitro by the DPPH free radical scavenging assay.

## 2. Results and Discussion

The recent applications of the biologically synthesized AuNPs showed promising advances for biomedical applications (i.e., acting as therapeutic agents—especially for cancer and drug delivery agents) [29]. Even though there are limited reports describing in vitro cyto- and hemocompatibility [30], based on this state-of-the-art condition, this study represents a stepping stone taking plant extracts with strong antioxidant behavior not only for the reduction of the Au metal salt but also for the formation of b-AuNPs, with the conservation of most of the functional groups of these extracts, thus offering increased stability. The methodology used for b-AuNPs synthesis is based on a 0.01 wt% HAuCl_4_ aqueous solution to which various concentrations of aqueous plant extract solutions are added, as described under the Materials and Methods section.

### 2.1. Characterization of the b-AuNPs

#### 2.1.1. UV–Vis Spectroscopy

**Figure 1:**
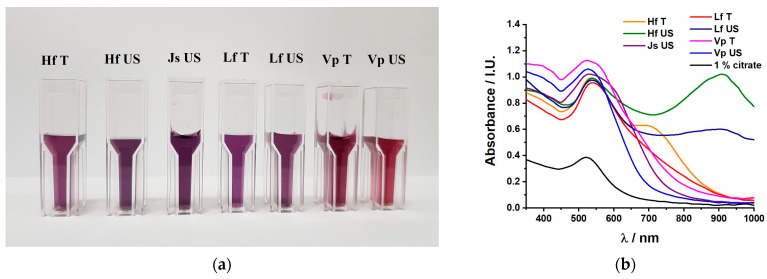
B-AuNP colloidal solutions of pressure enhanced (T) and ultrasound-assisted (US) sea buckthorn extracts (HfT and HfUS); lavender extracts (LfT and LfUS); grape extracts (VpT and VpUS) and ultrasound-assisted walnut extract (JsUS): (**a**) Picture of the as-prepared b-AuNP colloidal solutions; (**b**) UV–Vis absorption spectra of citrate-reduced AuNP (1% citrate) and b-AuNPs from (**a**).

UV–Vis spectroscopy provided quite a complex picture of the formation of b-AuNPs and insights into their morphology. The UV–Vis absorbance spectra confirmed the formation of AuNPs and b-AuNPs in Figure 1b. This also corresponded to a color change from a light yellow (aqueous HAuCl_4_ solution) to purple; Figure 1a. Depending on the various plant extracts, the reduction of gold was slightly different, resulting from the various shades of purple. Depending on the shape and position of the absorption maxima around 530 nm, characteristic of spherical AuNP nanoparticles [30], some morphological characteristics of the AuNPs may be emphasized. Sharp, well-defined absorption peaks, like in the case of citrate, VpUS, and LfT, suggest the formation of rather spherical, less monodispersed NPs [31]. In the case of LfUS, HfUS, and even HfT, a second absorption maximum at higher wavelengths (906 nm for both LfUS and HfUS, respectively, 705 nm for HfT) suggested the formation of anisotropic, rather polydisperse NPs [32]. The first stage of forming b-AuNPs corresponded to a sharp UV–Vis absorption maximum [33]. From the moment of extract addition, the color changed in a couple of seconds (LfUS), reaching a maximum of 5 min (HfUS). After that, the heating was turned off, and all solutions were left to cool at room temperature for another hour. No other change in the color or the absorption intensity was further observed. Previous work [26] showed that the LfUS extract has very high reducing activity. This has also been confirmed by a fast color change in the chloroauric solution.

**Figure 2:**
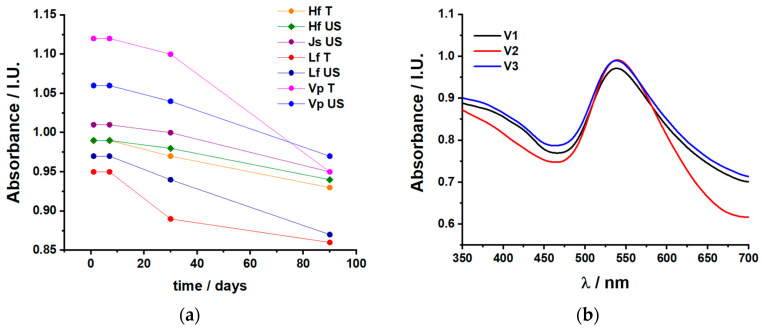
UV–Vis absorption spectra: (**a**) as a function of time dependency for all b-AuNPs (pressure-enhanced (T) and ultrasound-assisted (US) sea buckthorn extracts (HfT and HfUS); lavender extracts (LfT and LfUS); grape extracts (VpT and VpUS) and ultrasound-assisted walnut extract (JsUS)); (**b**) reproducibility of the b-AuNPs obtained from ultrasound-assisted sea buckthorn extract (HfUS).

For all spectra, the absorption maxima centered between 520 and 540 nm confirm the formation of AuNPs [30]. To tackle concerns such as stability and aggregation/sedimentation, Figure 2a shows spectra collected in time for all b-AuNPs by monitoring for 3 months (90 days) the intensity of the AuNP absorption maximum. The most stable, i.e., those corresponding to absorption maxima with the lowest decay, were collected for the HfUS, JsUS, VpUS, and HfT extracts. Based on visual observations as well, we can partially correlate the time stability with aggregation/sedimentation for the b-AuNP colloids obtained from HfT, LfUS, and VpT extracts. Reproducibility of the same b-AuNP solution is also a major concern; thus, Figure 2b highlights the absorption spectra of three different batches of HfUS-reduced b-AuNPS, following the same procedure. Although the shape of the absorption peak slightly differs (intensity and bandwidth), the maxima of the absorption wavelength are constant at 539 nm. However, the absorption maximum intensity and width differences may suggest differences in size distribution.

Further optimization steps were undertaken by varying both the metal salt concentration and the plant extract concentration. Thus, the b-AuNPs concentration was varied for 0.01%, 0.02%, and 0.03% using the 0.5 mL HfUS extract as a reducing agent, while the HfUS concentration varied from 0.25 mL to 1.0 mL in a final volume of 12 mL, representing 2%, 4%, 6%, and 8% of the extract to obtain a solution of 0.01% b-AuNPs. Absorption spectra for both varied parameters are presented in Figure S1. (Supplementary Materials). The optimal concentration for the metal salt was found to be 0.01%, where the optimum plant extract concentration was 4% (0.5 mL). A concentration of 2% (0.25 mL) did not show enough reducing behavior (very small intensity of the absorption maximum). In comparison, 6% (0.75 mL) or 8% (1 mL) no longer showed the presence of the second absorption maximum at 906 and 804 nm, respectively, for the tested HfUS extract. Both extract composition and concentration influence the shape and size of the NPs. Thus, for high concentrations of HfUS, the triangle shapes (highlighted through TEM measurements) are no longer formed.

Based on these observations, b-AuNPs prepared via HfUS, VpUS, LfT, and JsUS extracts were further investigated by TEM and FTIR analysis.

#### 2.1.2. Transmission Electron Microscopy

**Figure 3:**
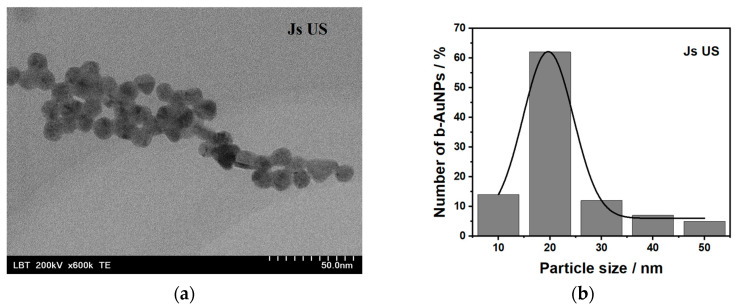

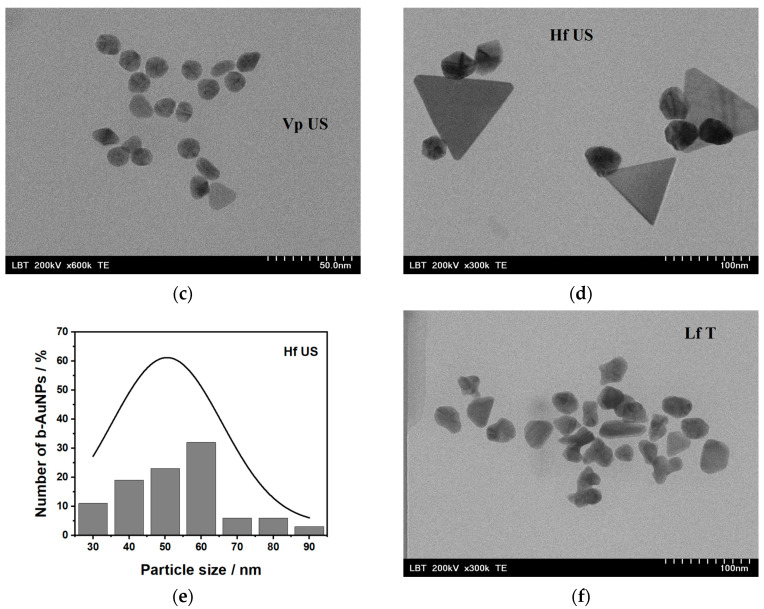
TEM images for b-AuNPs were obtained using plant extracts (**a**) JsUS, (**c**) VpUS, (**d**) HfUS, and (**f**) LfT, with corresponding size distribution histograms of b-AuNPs diameter for (**b**) JsUS and (**e**) HfUS with inset of the Gaussian distribution fit.

The shape and size of b-AuNPs were determined using TEM for four colloidal solutions obtained by reduction with JsUS (walnut), VpUS (grape), HfUS (sea buckthorn) and LfT (lavender) extracts, illustrated in Figure 3a,c,d,f, alongside two size distributions for b-AuNPs from JsUS and HfUS in Figure 3b,e. For comparison, chemically synthesized AuNPs (1% citrate) are shown in Figure S2. Except for b-AuNPs from HfUS, where both spherical and triangular shapes were observed, all other samples had a spherical shape and showed good dispersibility. The diameter of the spherical b-AuNPs in HfUS (Figure 3e) varied in a wide range from 28 to 96 nm, with an average size of 50.5 ± 2.3 nm. The heights of the triangular-shaped structures (significantly lower in numbers) could be averaged around the 175 nm edge length, three times bigger in size compared to the spherical NPs. LfT reduced b-AuNPs showed rather irregular shapes. B-AuNPs reduced with the grape extract (VpUS) presented quite a narrow distribution with diameters ranging between 8 and 34 nm and an average size of 20.0 ± 0.01 nm. A similar behavior was observed for the JsUS-reduced b-AuNPs, where the diameters ranged between 17 and 59 nm with an average value of 19.6 ± 0.3 nm. For the AuNPs prepared using the classical chemical route, the diameters ranged between 21 and 63 nm with an average size of 38.3 ± 3.6 nm (Figure S2.)

Overall, all samples showed good dispersibility; agglomeration/aggregation was only observed in the case of AuNPs (citrate). All b-AuNPs were polydisperse except those prepared using the walnut extract (JsUS). Hence, 63% of the analyzed particles had an average diameter of 19.6 nm. The broad absorbance maximum also confirmed the roughly spherical monodispersed nanoparticle shape at 528 nm. For the b-AuNPs, the rather sharp, well-defined absorption maxima at 525 and 540 nm prepared via VpUS and LfT should hint towards less monodispersed particles but still with a spherical shape [32]. However, this has only been confirmed for VpUS-reduced b-AuNPs that showed irregular shapes in the TEM pictures (anisotropy). For HfUS-reduced b-AuNPs, both anisotropies as well as polydispersity are best highlighted. The absorbance maximum at 538 nm indicated the presence of spherical particles. A second absorption maximum at 906 nm suggested the presence of clearly defined triangles. Triangle shapes were observed in the literature at various wavelengths, depending on their size and reducing agent. For an edge length up to 117 nm, a sharp absorption peak was observed at 700 nm for a high yield of triangular NPs [34]. Large triangular shapes (between 0.05–18 µm) were also observed in the NIR region, with a broad absorption maximum of around 1300 nm [35].

#### 2.1.3. Fourier Transform Infrared Analysis

**Figure 4:**
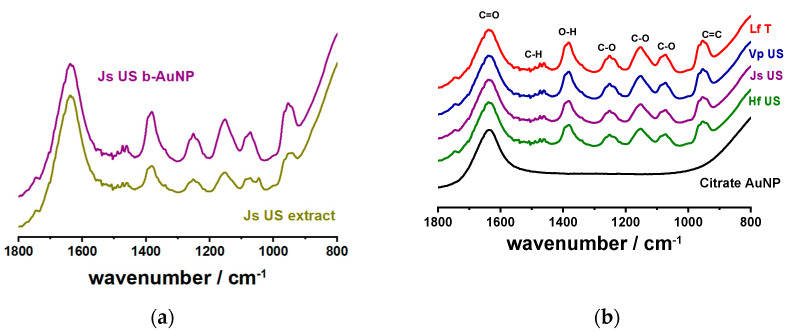
The FTIR-ATR spectra of the fingerprint region 1800-800 cm^−1^ for (**a**) walnut plant extract (JsUS) and AuNPs reduced by this extract; (**b**) several samples of gold nanoparticles prepared using plant extracts (pressure enhanced lavender extract -LfT, ultrasound-assisted sea buckthorn -HfUS, grape -VpUS and walnut -JsUS extracts).

The shape of FTIR-ATR spectra provided information about the functional groups transferred from the extracts to the b-AuNPs surface. It is well known that polyphenols exhibit antioxidant properties, having the capability to reduce chloroauric acid to AuNPs and stabilize the newly formed nanoparticles [10]. The fingerprint spectral region between 800 and 1800 cm^−1^ is monitored. Figure 4a highlights the similarities of the JsUS extract as such and its functional groups transferred to the b-AuNPs after reduction. Only the band at 1045 cm^−1^ (C-C stretching) disappeared from the spectra of b-AuNPs. Figure 4b depicts the spectra of b-AUNPs prepared to take the different plant extracts compared to citrate-reduced AuNPs. All the selected extracts contained antioxidant compounds, mainly phenolic compounds, as highlighted by the O-H bending at 1382 cm^−1^ and C-O bending and stretching vibrations in the spectral range between 1249 and 1073 cm^−1^ [36]. The C-O vibration at 1249 cm^−1^ highlights the stretching of primary alcohols [37], while the C-O stretching at 1156 and 1073 cm^−1^ suggests the presence of aliphatic ethers and primary alcohol flavonoids [38]. The weaker signal at 1497 cm^−1^ (C-H bending) can be assigned to the presence of flavonoids, while the C-C stretching at 953 cm^−1^ highlights the presence of aromatic compounds [36]. The C=O stretching at 1636 cm^−1^ is characteristic of amide bonds [39]. In the literature, there is also spectral evidence of aromatic ring stretching (C=C) around that wavenumber [40]. However, we suppose the presence of amides in our extracts is more pronounced than that of alkenes. This spectral evidence demonstrates that the b-AuNPs synthesized in this work retain the antioxidant compounds from the plant extract used for the reduction. These functional groups may scavenge ROS, such as H_2_O_2_, or free radicals, such as DPPH, as shown in Section 2.2.

### 2.2. Catalytic and Antioxidant Properties of the b-AuNPs

Electrochemical measurements were performed to highlight the catalytic and antioxidant properties of the b-AuNPs towards H_2_O_2_. To elucidate the catalytic properties, carbon-based SPEs were modified with b-AuNPs, and CV studied how H_2_O_2_ influences their redox behavior. H_2_O_2_ may cause lipid peroxidation, significantly affecting our health. Thus, DPPC lipid model membranes were prepared in the presence and absence of b-AuNPs, after which their behavior under the action of H_2_O_2_ was analyzed using CV and EIS measurements. Additionally, the antioxidant activity of b-AuNPs was validated by amperometry (with the help of the same AuNP-modified carbon-based SPE) and the DPPH free radical scavenging assay.

#### 2.2.1. Catalytic Properties

**Figure 5:**
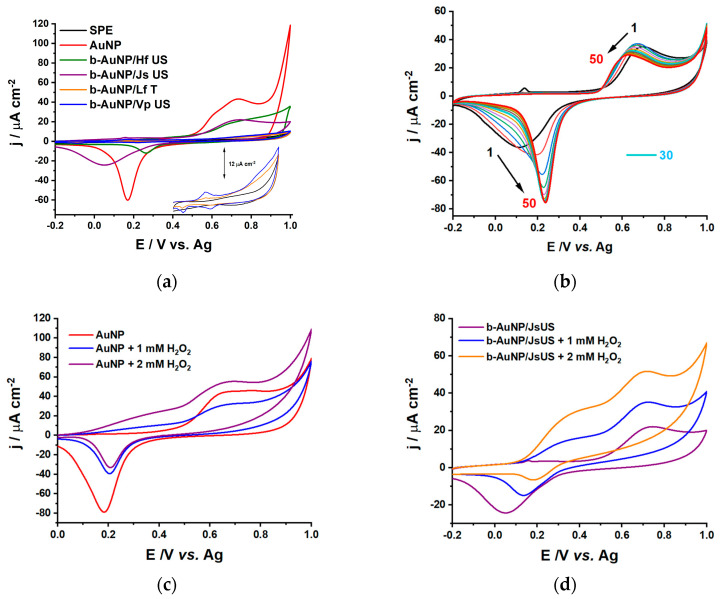
CVs in 0.1 M NaPB, pH 7.0, scan rate 50 mV s^−1^, step 2 mV for (**a**) bare SPE and modified SPE with AuNP and b-AuNP (using pressure-enhanced lavender extract -LfT, ultrasound-assisted sea buckthorn -HfUS, grape -VpUS and walnut -JsUS extracts); (**b**) Stability for Js US reduced b-AuNP modified SPE; (**c**) AuNP modified SPE in the absence and presence of 1 and 2 mM H_2_O_2_ and (**d**) b-AuNP/Js US modified SPE in the absence and presence of 1 and 2 mM H_2_O_2_.

To assess the electrocatalytic properties of the b-AuNPs, bare carbon-based SPE was modified with the b-AuNP-based colloidal solutions via drop-casting and was analyzed using electrochemical techniques, such as cyclic voltammetry (CV). Commercially available AuNP-modified SPE electrodes typically exhibit catalytic properties [15]. In line with this, in all the modified sensors presented in Figure 5a, redox behavior showed an oxidation peak in the range of 0.70–0.75 V and a corresponding reduction peak around 0.2 V for CVs recorded in 0.1 M sodium phosphate buffer (NaPB) between −0.2 and 1.0 V vs. Ag. b-AuNP/JsUS was an exception, for which the reduction of gold occurred at 0.05 V. Compared to the bare carbon-based SPE, which showed no redox behavior (inset in Figure 5a), gold nanoparticles deposited on the electrode surface showed electrocatalytic activity. The differences in current intensity can be attributed to the functional groups present on the b-AuNPs and their surface areas.

High current densities and reversible redox peaks highlight the electrocatalytic effect of most sensor configurations. The redox peaks have a small intensity for b-AuNPs obtained from LfT and VpUS extracts. From the TEM imaging, we know that b-AuNPs from LfT present irregular shapes, decreasing the available surface area compared to spherical NPs. The average sizes of the NPs also play an important role, citrate-reduced AuNPs being almost twice as large compared to VpUS and JsUS-reduced b-AuNPs; thus, higher current densities were recorded. Even though FTIR measurements showed similar fingerprints for all b-AuNPs, their catalytic behavior and the scavenging properties differed. This may be associated with the differences among the antioxidant compounds present in the extracts, which may be associated with the weak bands in the ATR-FTIR spectra. The variation in the current density of each sensor is shown in Table 1.

In addition, due to the good reversibility of the AuNP redox behavior, the b-AuNP/JsUS modified sensor was selected for further investigations. To assess the stability of the b-AuNPs on the sensor surface (Figure 5b), 50 consecutive CVs in NaPB, at a pH 7.0, with a scan rate of 50 mV s−1, between −0.2 and 1.0 V, were collected. After 30 scans, the shape of the voltammograms indicated a quasi-reversible behavior. A shift of the oxidation peak was observed from 0.7 to 0.6 V, with a current density decrease from 35.3 to 29.0 µA cm^−2^. Similarly, a shift of the reduction peak was also observed from 0.1 to 0.2 V, with a current density increase from −36.0 to −75.5 µA cm^−2^. These parameters are comparable to commercially available SPE-containing gold NPs [15]. In the same conditions, 50 consecutive CVs with 2 mM H_2_O_2_ were also collected (Figure S3a). Changes in the oxidation peak current density can be observed, with an increase of 42 µA cm^−2^ from the first to the tenth scan, after which it slightly decreases and stabilizes after the 30^th^ scan, with a slight shift of the peak potential towards lower values (from 0.65 to 0.62 V). For the reduction peak, no significant change occurs. Thus, we can conclude that H_2_O_2_ undergoes an irreversible oxidation catalyzed by the JsUS-modified b-AuNPs. Compared to Figure 5d and Figure S4, where the reduction peak completely disappears with increasing H_2_O_2_ concentrations, we can assign this peak as an intrinsic characteristic of the gold core of the b-AuNPs and not to their functional groups, which act as capping agents and their behavior strongly depends on the presence of H_2_O_2_ (H_2_O_2_ scavenging due to antioxidant properties). After the 50 scans, the electrode was washed, and a single CV was recorded in fresh 0.1 M NaPB at pH 7.0 to observe whether any changes occurred on the electrode surface (Figure S3b). No change was observed in the cathodic region, while in the anodic region, a 20 mV shift towards higher potentials and an increase in current density of 6 µA cm^−2^ could be observed. Thus, we can conclude that the b-AuNPs on the electrode surface demonstrate high stability.

Figure 5c,d discloses the catalytic behavior of the chemical and biologically synthesized AuNPs in the presence of H_2_O_2_. AuNP and b-AuNP/JsUS-modified SPE CVs were recorded before and after H_2_O_2_ addition (1 and 2 mM). Both modified sensors provided clearly defined redox profiles. After adding H_2_O_2_, the shape of the voltammograms changed depending on the characteristics of AuNPs. For chemically synthesized AuNPs (Figure 5c), the intensity of both AuNPs redox peaks at 0.2 and 0.65 V decreased for a lower concentration of H_2_O_2_. Increasing the concentration, the AuNP reduction peak keeps decreasing while the oxidation peak increases. For the second concentration of H_2_O_2_, an additional, small oxidation peak appears at 0.35 V. In literature [41], it was shown that on a clean gold surface, the oxidation of H_2_O_2_ provides only one peak around 0.4–0.5 V. In the presence of impurities or other materials (in our case, the carbon-based SPE, which was modified with the AuNPs), this peak shifts towards higher potentials around 0.7–0.8 V, overlapping with the oxidation potential of gold. Since the reduction peak remained almost unchanged, we can assign this as an intrinsic characteristic of the AuNPs. The JsUS-synthesized b-AuNPs showed a different behavior (Figure 5d). Similar to the chemically synthesized AuNPs, the first oxidation peak at 0.35 V is already visible for the first H_2_O_2_ concentration, and the current intensity keeps increasing with increasing H_2_O_2_ concentration. The b-AuNP oxidation peak current at 0.7 V keeps increasing, while the reduction peak decreases with a shift of the potential towards higher values (from 0.05 V to 0.2 V). Gradually, with the increase in H_2_O_2_ concentrations, the reduction peak completely disappeared (Figure S4), suggesting an irreversible oxidation of H_2_O_2_. The disappearance of the b-AuNPs reduction peak in the presence of H_2_O_2_ hints towards the scavenging properties of the functional groups (antioxidants) present on the AuNPs surface, which donate the hydrogen from their active hydroxyl groups and generate stable radicals [15], hindering AuNP reduction.

To further assess the catalytic effect taken up by the chemically and biologically synthesized AuNPs in the presence of 2 mM H_2_O_2_, the percentage of electrocatalytic uptake was calculated. Thus, for the AuNPs in Figure 5c, a decrease of 58% was observed for the reduction peak current, whereas an increase of 123% was observed for the oxidation peak current. JsUs-modified b-AuNPs (Figure 5d) showed a higher decrease of 73% for the reduction peak current. This difference in decrease is due to the H_2_O_2_ scavenging in the presence of b-AuNPs. In the case of the oxidation peak current, an increase of 229% was calculated, highlighting that b-AuNPs significantly improve the electron transfer, enhancing the electrocatalytic behavior of H_2_O_2_. Furthermore, variation of the scan rate controls how fast the applied potential is varied and, consequently, how fast the electron transfer occurs. Figure S5 shows the scan rate variation for the b-AuNP/JsUS modified electrode in the absence and presence of 2 mM H_2_O_2_. In the absence of H_2_O_2_, there is a considerable increase in the redox peak currents with increasing scan rate. The oxidation peak potential (0.65 V) is almost unchanged, with the peak current exhibiting an increase with increasing scan rates. A slight shift to the left of the sharp reduction peak potential can be observed from 0.26 V to 0.22 V, concluding that the two peaks characterize the b-AuNP redox and its electrocatalytic capacity. In the presence of H_2_O_2_, there is a considerable increase in the oxidation peak currents with increasing scan rates and a slight shift of the oxidation potential from 0.63 to 0.67 V, while the reduction peak slowly starts forming with increasing scan rates. We can emphasize that b-AuNPs significantly improve the irreversible oxidation of H_2_O_2_ through an improved electron transfer, while the scavenging properties of the antioxidant groups covering the AuNPs slowly decrease with increasing scan rates due to their oxidation.

#### 2.2.2. Antioxidant Properties

**Figure 6:**
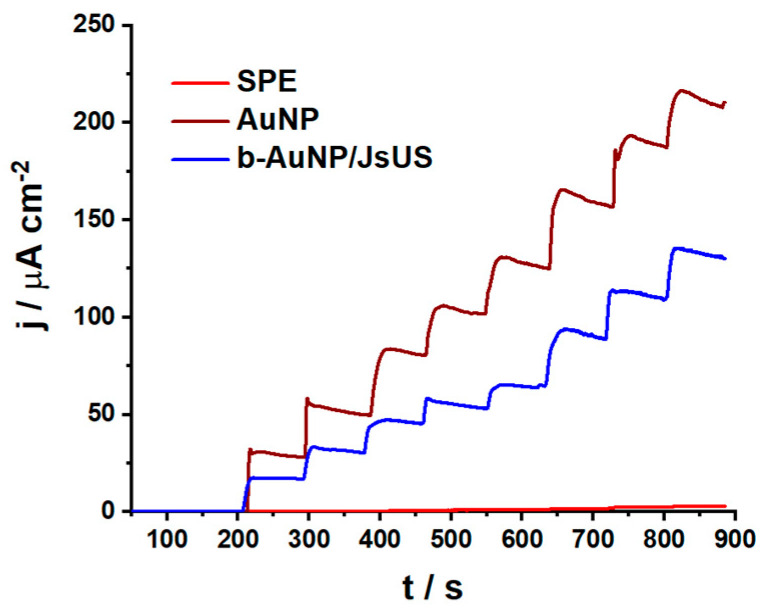
Amperometry at 0.65 V vs. Ag for H_2_O_2_ sensors: SPE, AuNP, and b-AuNP/JsUS (ultrasound-assisted walnut extract), in 0.1 M NaPB, pH = 7.0.

Several techniques have been employed to assess the scavenging effect of the b-AuNPs on H_2_O_2_. First, the AuNP-modified carbon-based SPE can be studied using fixed-potential amperometry. It is known that H_2_O_2_ presents a characteristic oxidation peak at the potential of 0.6 V15; thus, a potential of 0.65 V vs. Ag in 0.1 M NaPB pH 7.0 was chosen for amperometry measurements in Figure 6 where the response of three SPEs is highlighted: the unmodified electrode, AuNP, and b-AuNP/JsUS-modified electrodes, in time for the incremental concentration of H_2_O_2_ (from 1 to 11 mM). A weaker response to H_2_O_2_ can be observed for the b-AuNP/JsUS-modified sensor, highlighting the scavenging potential of the biologically synthesized AuNPs. The amperometries can be further analyzed in terms of sensor sensitivity (S) and limit of detection (LoD = 3 × SD/S, where SD represents the standard deviation of the blank), which were determined for triplicate measurements in the 1–5 mM linear range and are shown in Figure S6a. For AuNP, a sensitivity of 29.85 ± 2.01 µA cm^−2^ mM^−1^ with a LoD of 0.64 mM was measured. For the b-AuNP/JsUS and b-AuNP/HfUS sensors, both sensitivity and LoD were decreased (S = 18.79 ± 0.93 µA cm^−2^ mM^−1^ and LoD = 0.47 mM for the first and S = 18.64 ± 1.16 µA cm^−2^ mM^−1^ and LoD = 0.59 mM for the second, Figure S4b). A lower sensitivity (decreased by 37% for both b-AuNPs) corresponded to a scavenging effect towards H_2_O_2_ due to the functional groups on the b-AuNPs.

**Figure 7:**
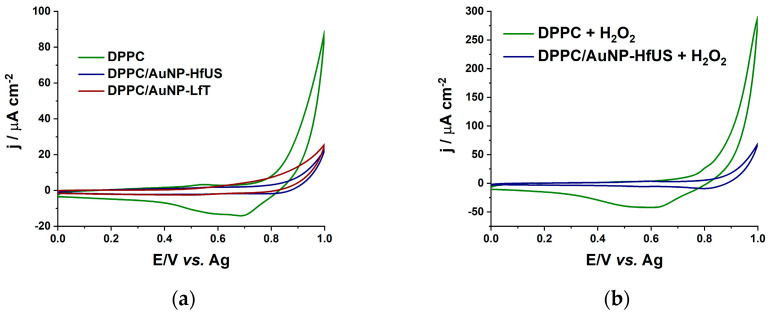
CVs in 0.01 M PBS, pH 7.4, scan rate 50 mV s^−1^, step 2 mV for (**a**) all DPPC liposomal systems (DPPC, DPPC/AuNP-HfUS (ultrasound-assisted sea buckthorn extract) and DPPC/AuNP-LfT (pressure-enhanced lavender extract)); (**b**) DPPC and DPPC/AuNP-HfUS in the presence of 3 mM H_2_O_2_.

The study of nanoparticle interaction with biological cells and penetration into cell membranes may have crucial implications for the nanoparticles’ biomedical applications [42]. Thus, the DPPC liposomal system was chosen as the cell membrane model. Further, increased H_2_O_2_ concentrations affect biological processes such as cytotoxic effect, apoptotic effect, or oxidative stress [43]. Thus, the effect of b-AuNPs in DPPC liposomal systems in the absence and presence of H_2_O_2_ was analyzed using CV and EIS measurements. Figure 7a shows the CVs in PBS of the DPPC liposomal systems formed in the presence and absence of b-AuNPs recorded at a gold chip surface. For the DPPC/b-AuNPs system, a decrease in the CVs width can be noticed, suggesting a decrease in electrical capacitance at the chip surface by modifications of lipid system properties due to interactions between the liposomes facilitated by the presence of b-AuNPs integrated into liposome membranes. Figure 7b shows the DPPC and DPPC/AuNP-HfUS liposomal system CVs at the chip surface in PBS with 3 mM H_2_O_2_. For the DPPC liposomal system, a broad reduction peak of around 0.6 V (shifted from 0.7 V in the absence of H_2_O_2_) and a pronounced capacitive behavior appeared upon H_2_O_2_ addition. This may suggest lipid peroxidation in the presence of H_2_O_2_, leading to an increase in the system’s capacitance. For the DPPC/AuNP-HfUS system, however, a small oxidation peak is visible around 0.6 V, highlighting the oxidation of H_2_O_2_ at b-AuNPs, suggesting that the nanoparticles are integrated in the lipid membrane. Given the electrocatalytic properties of b-AuNPs and the isolating nature of lipids, we can assume that the b-AuNPs are in the lipid membrane, and any change in the voltammogram is due to the presence of H_2_O_2_. The changes in the capacitive behavior in the CVs obtained in the presence of H_2_O_2_ for the DPPC/AuNP-HfUS and DPPC/AuNP-LfT systems are significantly reduced compared to DPPC alone, highlighting the scavenging effect of b-AuNPs (Figure S7a–c) and promoting lipid protection from peroxidation of b-AuNPs towards H_2_O_2_.

**Figure 8:**
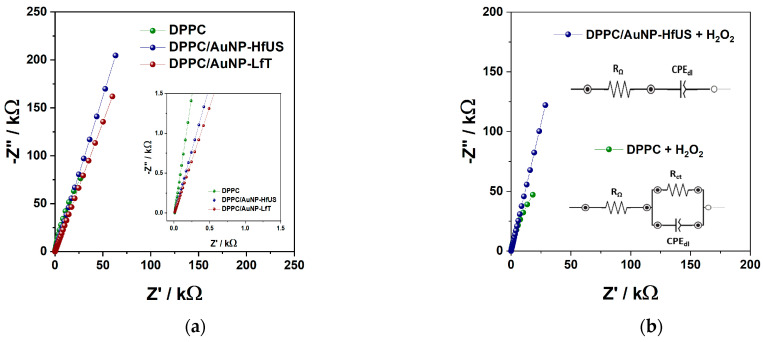
Nyquist spectra (EIS) in 0.01 M PBS, pH 7.4, applied potential of +0.1 V vs. Ag for (**a**) all DPPC liposomal systems (DPPC, DPPC/AuNP-HfUS (ultrasound-assisted sea buckthorn extract) and DPPC/AuNP-LfT (pressure-enhanced lavender extract)); (**b**) DPPC and DPPC/AuNP-HfUS in the presence of 3 mM H_2_O_2_.

To better emphasize the interactions of liposomal systems, b-AuNPs, and H_2_O_2_, EIS spectra were also recorded in the 0.1 Hz–20 kHz frequency region. These highlight the electron transfer and H_2_O_2_ diffusional processes. Figure 8 shows the complex plane representation of the impedance spectra recorded at a potential of 0.1 V vs. Ag, chosen in the non-faradaic region of the CVs. The spectra were fitted with an equivalent electrical circuit of resistors and capacitors in the intermediate and low-frequency regions (Au-chip/liposomal system interface). The high-frequency region was omitted during fitting due to its increased capacitive behavior (inset Figure 8a). Two circuits were used to fit the spectra, depending on the liposomal system, and they are presented in Figure 8b. For DPPC alone, a cell resistance is in series with a parallel combination of a charge transfer resistance (R_ct_) and a pseudo-capacitance represented as a constant phase element (CPE_dl_) which is modeled as a non-ideal capacitor with impedance: Z_CPE_ = [(Ciω)^α^]^−1^, where C is the ideal capacitance, ω is the radial frequency, and the exponent α reflects the deviation from a regular capacitor. For the presence of b-AuNPs in the liposomal system, the circuit is simplified to a CPE_dl_ in series with the cell resistance (R_Ω_). This suggests that the b-AuNPs are incorporated (charge accumulation) in the membrane (DPPC/b-AuNPs), increasing the capacitance of the systems (6.799 µF s^α−1^ compared with 0.179 µF s^α−1^ for DPPC). In all systems, after the addition of 3 mM H_2_O_2_, the impedance of the system decreases for low frequencies (0.1 Hz) (Figure S8a–c). The presence of b-AuNPs has an influence on this change: for DPPC alone, the capacitance of the system increases in the presence of H_2_O_2_, while R_ct_ decreases (Table S1), highlighting the influence of H_2_O_2_ on the liposomes by restructuring the liposomal membranes due to lipid peroxidation which may cause the appearance of pores facilitating charge transfer to the chip surface; in the presence of b-AuNPs, the capacitance of DPPC/b-AuNPs systems decreases, and R_ct_ increases to such extent that it cannot be fitted (especially in the high-frequency region), hindering the influence of H_2_O_2_ on the DPPC/b-AuNPs because of H_2_O_2_ scavenging by the b-AuNPs. These results agree with the results obtained by cyclic voltammetry. To better highlight the changes in capacitance, the Bode phase plot is also represented in Figure S8d–f for all liposomal systems. In all cases, the Bode plot shows that the phase increased from −70 deg for low frequencies to −80 deg at intermediate ones, which corresponds to a capacitive behavior which again confirms the charge accumulation due to the liposomes. For high frequencies, for the DPPC liposomes alone, with increasing frequency, phase shift diminishes approaching −10 deg. The decrease is a little faster in the presence of H_2_O_2_, approaching 0 deg, which promotes the charge transfer (lipid peroxidation). For DPPC/b-AuNPs, in the low and intermediate frequencies, the phase remains constant at ~−73 deg, followed by a decrease towards ~−45 deg for high frequencies, highlighting the presence of conducting b-AuNPs (decreasing in capacitance and the increasing of the charge transfer due to ions from electrolyte). In the presence of H_2_O_2_, in the low and intermediate frequencies, the phase slightly increases but remains constant at ~−76 deg and decreases towards ~−15 deg for high frequency, highlighting the increase in the charge transfer (of H_2_O_2_ towards b-AuNP and electrons resulting in its oxidation). These values can also be correlated to the values of the α exponent, which decrease when b-AuNPs are integrated into liposome membranes (range between 0.774 and 0.853) compared with 0.899 for DPPC. The closer the value to one, the higher the system’s resemblance to the uniform surface of an ideal capacitor. When H_2_O_2_ is present in the DPPC system, the α value decreases to 0.885, indicating a porous, non-uniform liposomal membrane, again showing lipid peroxidation’s damaging effect. The opposite effect can be observed for DPPC/b-AuNPs systems when the α value increases.

**Figure 9:**
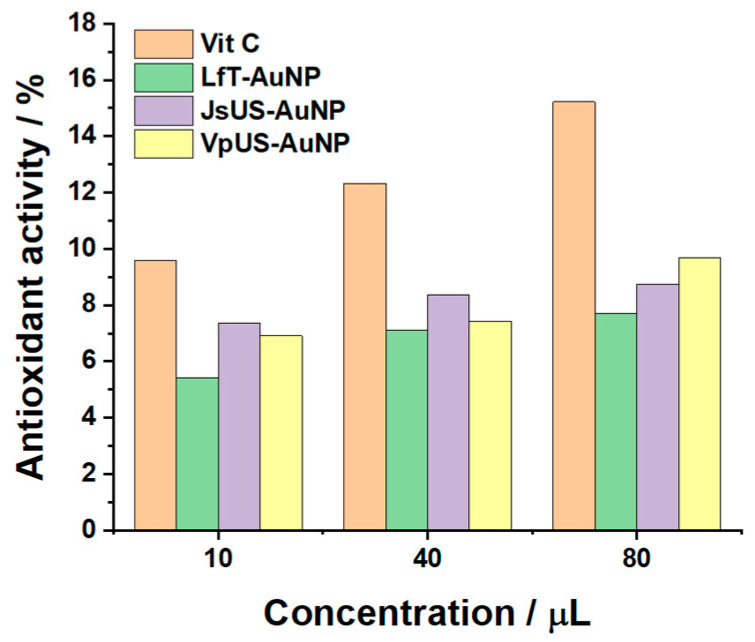
The in vitro antioxidant activity of pressure-enhanced lavender—LfT, ultrasound-assisted walnut—JsUS, and grape—VpUS reduced b-AuNPS in comparison with vitamin C.

To validate these results, Figure 9 highlights the free radical scavenging property of the b-AuNPs in comparison to vitamin C for DPPH. The antioxidant activity is increasing with increasing concentrations of both Vit C and b-AuNPs. Even if not so powerful as vitamin C, VpUS-reduced b-AuNPs still present an antioxidant activity of 72% (for 10 µL) and ~63% (for the next two concentrations) from Vit C. Thus, all b-AuNPs present antioxidant activity, where b-AuNP/VpUS seem to be generating the most potent chain reactions.

## 3. Materials and Methods

### 3.1. Materials

Gold (III) chloride trihydrate (HAuCl_4_ × 3H_2_O; 99.9%; MW = 393.83 g mol^−1^) and 2,2-Diphenyl-1-picrylhydrazyl (DPPH, MW = 394.35 g mol^−1^) were purchased from Sigma-Aldrich. The phospholipid dipalmitoylphosphatidylcholine (DPPC) with >99% purity was purchased from Avanti Polar Lipids and was a gift from Dr. Arkadiusz Matwijczuk. H_2_O_2_ (30%, MW = 34.02 g mol^−1^) and trisodium citrate dihydrate (C_6_H_5_O_7_Na × 2H_2_O; MW = 294.10 g mol^−1^) were purchased from SC. NORDIC INVEST SRL. Ethanol and chloroform of pharmaceutical grade and ascorbic acid (vit C, MW = 176.12 g mol^−1^) were obtained from S.C. Chemical Company S.A. (Iași, Romania) All measurements were performed in pH 7.0 sodium phosphate buffer (NaPB, 0.1 M) at room temperature (22 ± 1 °C). The phosphate buffer was prepared by using NaH_2_PO_4_ and Na_2_HPO_4_. For liposomal systems, 10 mM, pH 7.4 phosphate-buffered saline (PBS) tablets were used and purchased from Sigma-Aldrich. Millipore Milli-Q nanopore water (resistivity ≥ 18 MΩ cm) was used for the preparation of all solutions.

### 3.2. Preparation of Plant Extracts

The extracts were obtained through methods previously described [26]. Sea buckthorn berries (Hf), walnuts (Js), lavender flowers (Lf), and red grape skin and seeds (Vp) were collected from Romanian plants, dried, and crushed. The water of pH = 9.5 (obtained using a Leveluk Kangen water apparatus) was used for the extraction process. Then, water was mixed in a 1:1 ratio with ethanol, to which the powdered plant was added in a proportion of 1 g to 10 mL solvent, followed by extraction. Two extraction methods were used: ultrasound-assisted extraction (US) and pressure-enhanced solvent extraction at 6.7 bar (T).

### 3.3. Colloidal AuNP Solution Synthesis

Metallic gold nanoparticles (AuNPs) were synthesized using chemical routes according to Sanz et al. [44], where a final solution of 0.01% AuNPs was obtained using sodium citrate as a reducing agent. In accordance, 1 wt% of sodium citrate solution was slowly added under continuous stirring to a solution of 0.01 wt% HAuCl_4_ × 3H_2_O in water and then heated up to its boiling point. The heating was stopped when the color turned purple, and the solution was left to cool at room temperature under the same stirring conditions. The AuNP dispersion was then purified by centrifugation at 13,400 rpm for 20 min using an Eppendorf Minispin microcentrifuge. The solid residue was finally redispersed in Milli-Q water, sonicated for 5 min to ensure a homogeneous distribution, and kept in the refrigerator at 4–6 °C for further use.

Biologically synthesized gold nanoparticles were prepared similarly. A solution of 0.01 wt% HAuCl_4_ × 3H_2_O in water was heated to 70 °C. After temperature stabilization, the plant extracts were added dropwise, and the heat was stopped. Depending on the plant extract, the light yellow solution turned purple within seconds, up to a maximum of 5 min. After cooling the solution to room temperature, the residue was washed as previously. Special attention was given to the washing step, and after optimizing the synthesis protocol, it was found that only one washing step is enough to remove excess plant-extract molecules, as shown in Figure S9. During the optimization steps, both metal salt concentration (from 0.01% to 0.03%) and extract concentration (from 0.25 mL to 1.0 mL) were varied in a final volume of 12 mL. The preparation utilized seven different plant extracts, namely two sea buckthorn extracts using both extraction methods: HfT and HfUS, two grape extracts: VpT and VpUS, two lavender extracts: LfT and LfUS, and a walnut extract: JsUS.

### 3.4. Formation of Liposomes for In Vitro Evaluation of the H_2_O_2_ Scavenging Activity

The H_2_O_2_ scavenging activity of the b-AuNPs was determined on a DPPC liposomal system, according to Kluczyk et al. [45]. Liposomal systems were prepared using the thin-film hydration method. First, the lipids were dissolved in chloroform at a concentration of 0.05 M in glass tubes and were left to evaporate. Then, the samples were conditioned under vacuum for about 1 h to form a thin, homogeneous, solvent-free film. A PBS buffer, 0.01 M, pH 7.4, was added to the dry sample and the b-AuNPs to obtain a concentration of 0.37 mg DPPC and 0.01% b-AuNP/1 mL buffer. Samples were placed in a water bath at 45 °C for 5 min. Next, they were removed and shaken in a vortex mixer for 10 s before being placed again in the water bath to initiate the hydration process, resulting in three liposomal systems: DPPC, DPPC/AuNP-HfUS, and DPPC/AuNP-LfT.

### 3.5. In Vitro Validation of the Antioxidant Activity

The antioxidant activity of the b-AuNPs was determined by the DPPH free radical scavenging assay [46]. A 0.1 mM DPPH solution was prepared with ethanol, and absorbance was recorded at 519 nm. A total of 750 µL DPPH was mixed with 10, 40, and 80 µL of a 0.1 mM vitamin C stock solution (in ethanol) and brought to a final volume of 3 mL with ethanol. For 0.01% b-AuNPs colloidal solution, the same volumes as for vit-C were kept. The absorption maxima at 519 nm were recorded after an incubation time of 30 min. Scavenging of DPPH radicals was expressed as a percentage compared to the negative control without adding the sample (b-AuNPs), and vitamin C was used as a positive control. The antioxidant activity percentages were calculated as follows: % DPPH = [(A_0_ −A_1_)/A_0_] × 100, where A_0_ was the absorbance of the positive control (vit C) and A_1_ was the absorbance in the presence of the sample (b-AuNPs). For vitamin C, the control was DPPH.

### 3.6. Characterization Techniques

#### 3.6.1. UV–Vis Spectroscopy

The optical properties of the colloidal AuNP solutions were examined using a FLAME-S spectrometer (Ocean Optics Inc., Largo, FL, USA) preconfigured for 200–1050 nm. The balanced DH-2000-BAL deuterium tungsten halogen light source provides a 230–2500 nm illumination. Data were visualized and analyzed by the corresponding Ocean View 1.6.3 software. The path length for absorption spectra was 1 cm. The colloidal AuNPs were used as prepared. The absorption spectra of the b-AuNPs were measured on the same day of their synthesis and on subsequent days (up to 90 days) to demonstrate the stability of the colloids. Likewise, the spectra from the different batches were compared to assess reproducibility. All spectra are baseline subtracted; a feature provided by the data collection software.

#### 3.6.2. Transmission Electron Microscopy

Transmission electron microscopy (TEM) analysis was performed on a HITACHI HD-2700 STEM microscope (Hitachi, Japan) operating at 200 kV. Samples were deposited onto a carbon film on 400 mesh Cu grids. Images and dimensions were obtained using a Hitachi acquisition software, var. 8.1. a Gaussian fitting in Origin 2019b calculated the average particle size.

#### 3.6.3. Fourier Transform Infrared Spectroscopy

FTIR spectroscopy measurements were performed with a PerkinElmer Spectrum BX II apparatus (PerkinElmer Corporation, Waltham, MA, USA) in an attenuated total reflectance (ATR) mode using a Pike-MIRacle (PIKE Technologies, Madison, WI, USA) attachment with a diamond–zinc selenide crystal with a diameter of 1.8 mm. For each sample, a drop of 10 µL was placed on the top of the crystal, and the spectra were recorded in the transmission mode and transformed to absorbance for graphical representation. The spectra were registered to take 32 scans in the range of 500–4000 cm^−1^ with a resolution of 4 cm^−1^.

#### 3.6.4. Electrochemical Measurements

All electrochemical measurements were carried out in a 0.1 M NaPB, pH 7.0 buffer solution. Commercially available screen-printed carbon electrodes (SPEs) (DRP–110) deposited on a ceramic substrate with three silver electrical contacts connecting a carbon working electrode, a carbon auxiliary electrode, and a silver reference electrode (DropSens, Llanera (Asturias), Spain) were used. The working electrodes had a geometrical surface area of 0.1256 cm^2^. These were further modified by 2.5 µL drop-casting of colloidal AuNP and b-AuNP solutions in three successive steps with intermediate drying of 10 min. In vitro evaluation of H_2_O_2_ scavenging activity on a DPPC liposomal system was carried out in 0.01 M PBS, pH 7.4 saline buffer solution in a 100 µL electrochemical cell consisting of a glass chip covered with gold, acting as a working electrode (geometrical surface area of 1 cm^2^), a platinum wire as the auxiliary electrode, and a silver wire as reference electrode. A glass chip covered with gold (50 nm) was chosen to avoid liposome adhesion to the surface. Cyclic voltammetry, electrochemical impedance spectroscopy, and fixed potential amperometric measurements were performed using a PalmSens3 electrochemical sensor interface (Palm Instruments BV, Houten, The Netherlands) controlled by PSTrace 5.9 software.

## 4. Conclusions

This study focused on the biological synthesis of gold nanoparticles and their behavior in potential biomedical applications. Plant extracts with well-known antioxidant properties were used to synthesize the b-AuNPs biologically. Further, it was shown that the synthesized b-AuNPs have antioxidant properties, protecting against lipid peroxidation, highlighted through electrochemical methods using a DPPC liposomal system and validated by DPPH free radical scavenging assay. CV and EIS measurements suggest that the nanoparticles are integrated into the lipid membrane, and all changes in the spectra occur due to the damaging effect of H_2_O_2,_ promoting lipid peroxidation. The DPPH free radical scavenging assay showed a scavenging activity of 72% compared to Vit C. UV–Vis measurements and FTIR-ATR spectroscopy highlighted the presence of functional groups (especially -OH) on the b-AuNPs, which were associated with phenolic compounds and for which it is well known that they have powerful antioxidant activity.

Using label-free electrochemical sensors modified with both chemically synthesized and biologically synthesized AuNPs, the scavenging effect of the b-AuNPs upon H_2_O_2_ was demonstrated. For AuNP, a sensitivity of 29.85 µA cm^−2^ mM^−1^ was obtained, while for the b-AuNP modified sensors, the sensitivity towards H_2_O_2_ decreased (S = 18.79, 18.64 µA cm^−2^ mM^−1^). A lower sensitivity (37% decrease) corresponded to a scavenging effect. CV and EIS measurements also highlighted an improved catalytic behavior of the b-AuNPs.

## Figures and Tables

**Table 1:** AuNP redox peak current for all the modified CV sensors recorded in the −0.2–1.0 V window.

Sensor	Oxidation Peak Current/ μA cm^−2^	Reduction Peak Current/μA cm^−2^
SPE	-	-
AuNP	44.6	−61.5
b-AuNP/Hf US	20.7	−12.7
b-AuNP/Js US	22.1	−24.4
b-AuNP/Lf T	4.1	−0.6
b-AuNP/Vp US	5.2	−1.3

## Data Availability

The authors confirm that the data supporting the findings of this study are available within the article and its electronic Supplementary Materials. Raw data of this study are available from the corresponding authors, M.F. and C.B., upon reasonable request.

## Supplementary Materials

The following supporting information can be downloaded at https://www.mdpi.com/article/10.3390/ph17091105/s1, Figure S1: UV-Vis absorption spectra for: (a) AuNP concentration variataion obtained after using sea buckthorn extract (Hf US) as reducing agent; (b) Hf US plant extract concentration variation for 0.01% AuNP; Figure S2: (a) TEM image for citrate reduced AuNPs; Size distribution histograms of b-AuNPs diameter obtained using (b) citrate and (c) Vp US plant extract; with inset of the Gaussian distribution fit; Figure S3: CVs in 0.1 M NaPB, pH 7.0, scan rate 50 mV s^−1^, step 2 mV for (a) Stability for Js US reduced b-AuNP modified SPE in the presence of 2 mM H_2_O_2_, (b) b-AuNP/JsUS modified electrode before and after the 50 scans from (a); Figure S4: CV in 0.1 M NaPB, pH 7.0, 50 mV s^−1^, step 2 mV for SPE modified with b-AuNP obtained with JsUS extract, with increasing concentrations of H_2_O_2_ (1 to 5 mM); Figure S5: CVs in 0.1 M NaPB, pH 7.0, for b-AuNP/Js US modified electrode at different scan rates: 10, 20, 30, 50, 70, 90 and 110 mV s^−1^ (a) in the absence and (b) presence of 2 mM H_2_O_2_; Figure S6: (a) Calibration plots for AuNP and b-AuNP/JsUS (n = 3) corresponding to Figure 6 (manuscript); (b) Calibration plot for Hf US obtained b-AuNP modified sensor (n = 3) for increasing H_2_O_2_ concentrations in 0.1 M NaPB, pH = 7.0; inset: fixed potential amperometry at 0.65 V vs. Ag; Figure S7: (a) CVs in 0.01 M NaPBS, pH 7.4, scan rate 50 mV s^−1^, step 2 mV for all liposomal systems in the absence and presence of 3 mM H_2_O_2_ (a) DPPC, (b) DPPC/AuNP-LfT and (c) DPPC/AuNP-HfUS; Figure S8: EIS in 0.01 M PBS, pH 7.4, applied potential of +0.1 V vs. Ag in the absence and presence of 3 mM H_2_O_2_: Nyquist spectra of (a) DPPC, (b) DPPC/AuNP-LfT and (c) DPPC/AuNP-HfUS liposomal systems; Bode phase plots for (d) DPPC, (e) DPPC/AuNP-HfUS liposomal systems and (f) DPPC/AuNP-LfT; Figure S9: UV-Vis absorption spectra for: (a) diluted Hf US plant extract (4%), 0.01% HAuCl_4_ aqueous solution and washed 0.01% AuNP/HfUS colloidal solution. (b) magnification of (a) for 0.01% HAuCl_4_ aqueous solution and washed 0.01% AuNP/HfUS colloidal solution; Table S1: Equivalent circuit parameter values obtained by fitting the impedance spectra from Figure 8 and Figure S6.
