# Supplementary material for: Biologically Synthesized Gold Nanoparticles with Enhanced Antioxidant and Catalytic Properties
Source: Pharmaceuticals (Basel). 2024 Aug 23;17(9):1105. doi: 10.3390/ph17091105 (PMC11434865; doi:10.3390/ph17091105)
Supplement: Supplementary file 1 [file pharmaceuticals-17-01105-s001.zip › pharmaceuticals-3105719-supplementary.pdf]

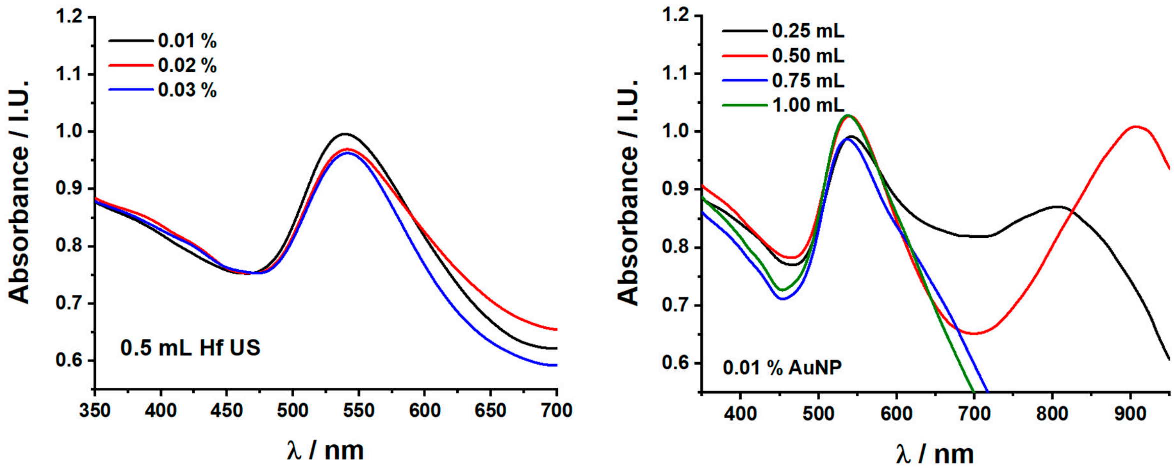

(**a**) (**b**)

Figure S1. UV-Vis absorption spectra for: (**a**) AuNP concentration variataion obtained after using sea buckthorn extract (Hf US) as reducing agent; (**b**) Hf US plant extract concentration variation for 0.01 % AuNP.

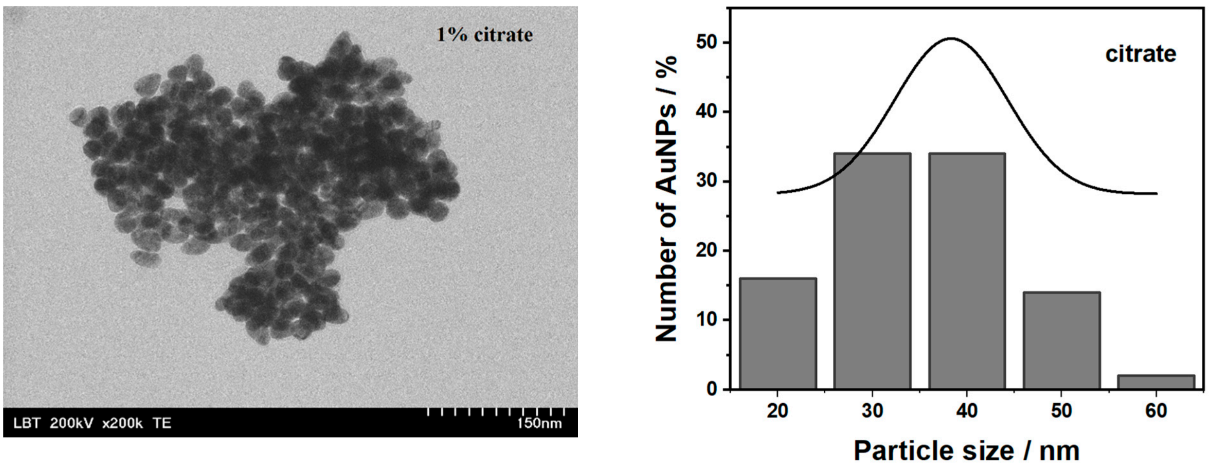

(**a**) (**b**)

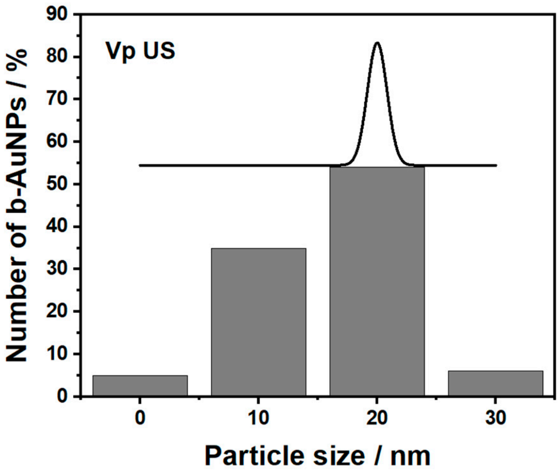

(**c**)

Figure S2. (**a**) TEM image for citrate reduced AuNPs; Size distribution histograms of b-AuNPs diameter obtained using (**b**) citrate and (**c**) Vp US plant extract; with inset of the Gaussian distribution fit.

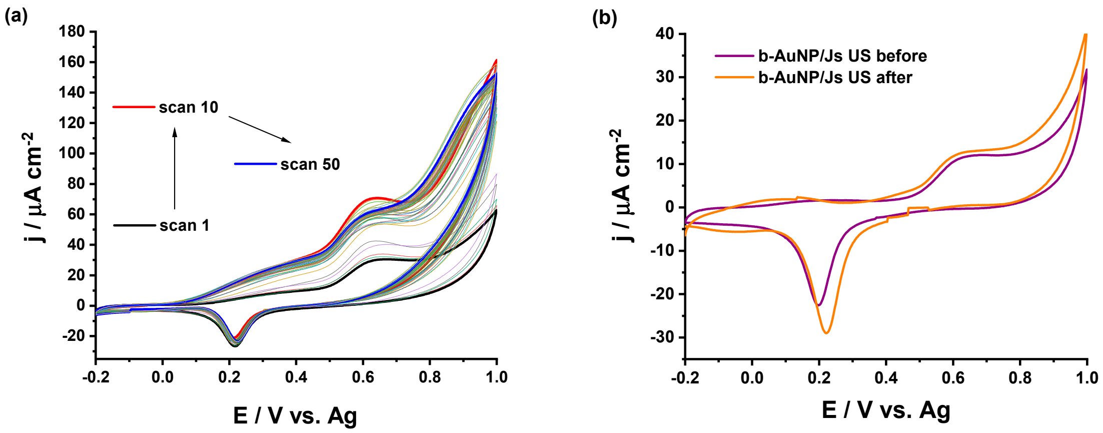

Figure S3. CVs in 0.1 M NaPB, pH 7.0, scan rate 50 mV s^{-1}, step 2 mV for **(a)** Stability for Js US reduced b-AuNP modified SPE in the presence of 2 mM H_2O_2, **(b)** b-AuNP/JsUS modified electrode before and after the 50 scans from (a).

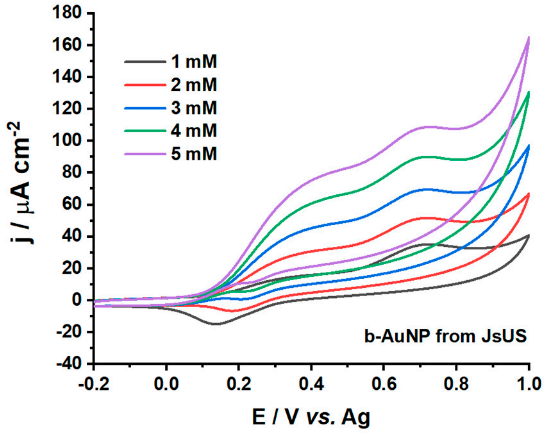

Figure S4. CV in 0.1 M NaPB, pH 7.0, 50 mV s^{-1}, step 2 mV for SPE modified with b-AuNP obtained with JsUS extract, with increasing concentrations of H_2O_2 (1 to 5 mM).

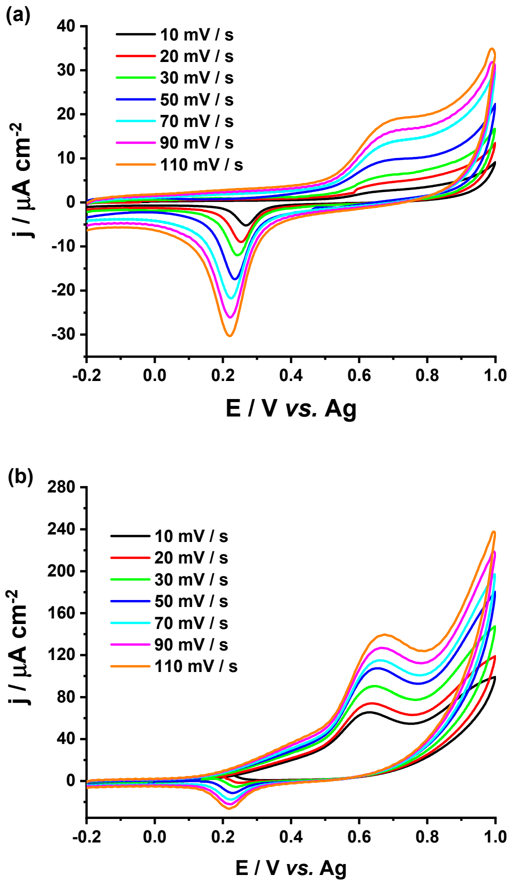

Figure S5. CVs in 0.1 M NaPB, pH 7.0, for b-AuNP/Js US modified electrode at different scan rates: 10, 20, 30, 50, 70, 90 and110 mV s^{-1} (a) in the absence and (b) presence of 2 mM H_2O_2.

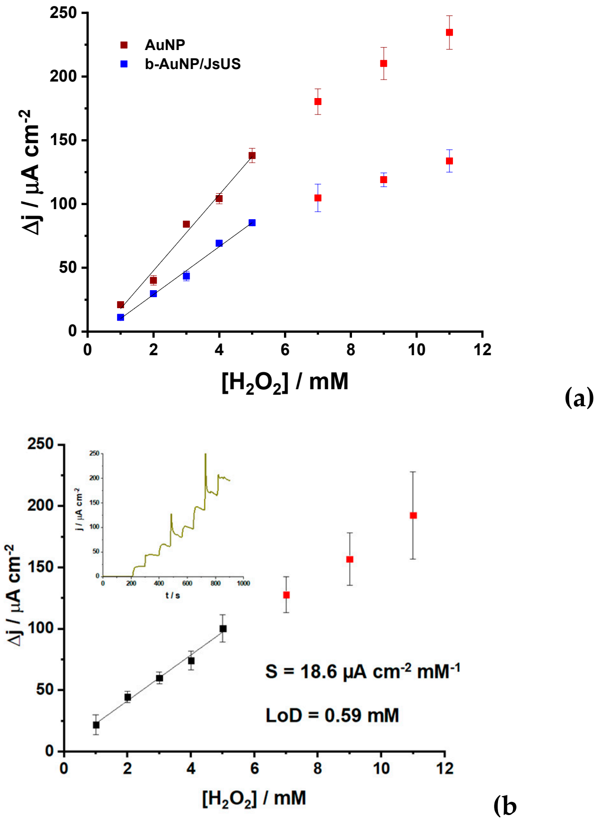

Figure S6. (a) Calibration plots for AuNP and b-AuNP/JsUS (n=3) corresponding to Fig 6 (manuscript); **(b)** Calibration plot for Hf US obtained b-AuNP modified sensor (n=3) for increasing H_2O_2 concentrations in 0.1 M NaPB, pH = 7.0; inset: fixed potential amperometry at 0.65 V vs. Ag.

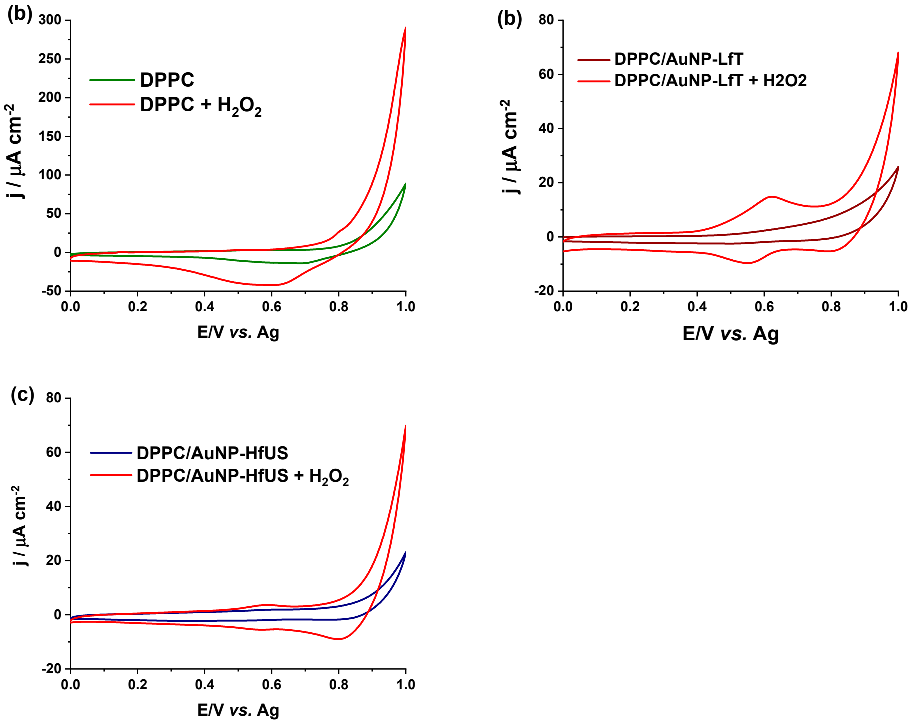

Figure S7. (a) CVs in 0.01 M NaPBS, pH 7.4, scan rate 50 mV s^{-1}, step 2 mV for all liposomal systems in the absence and presence of 3 mM H_2O_2 **(a)** DPPC, **(b)** DPPC/AuNP-LfT and **(c)** DPPC/AuNP-HfUS.

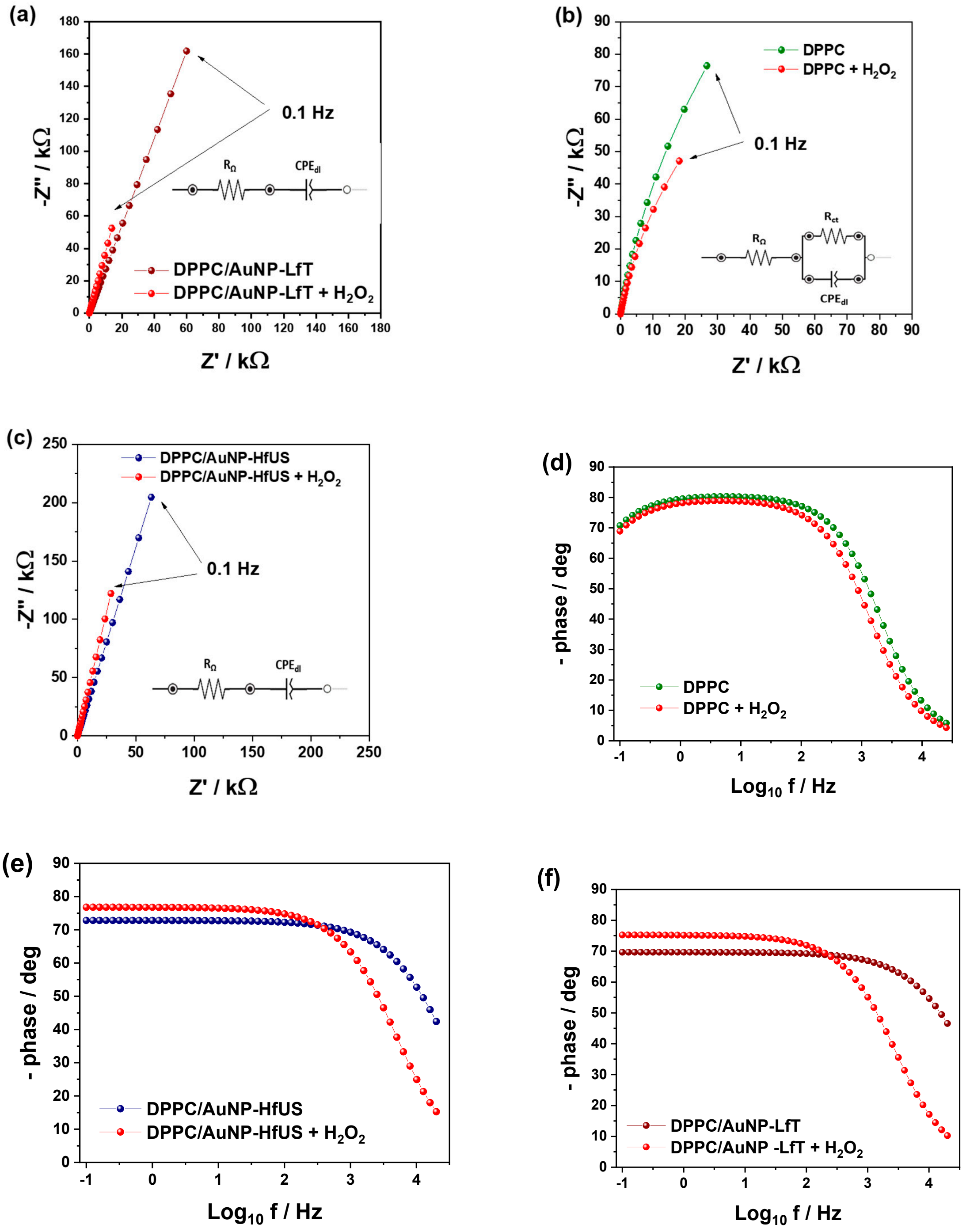

Figure S8. EIS in 0.01 M PBS, pH 7.4, applied potential of +0.1 V vs. Ag in the absence and presence of 3 mM H_2O_2: Nyquist spectra of **(a)** DPPC, **(b)** DPPC/AuNP-LfT and **(c)** DPPC/AuNP-HfUS liposomal systems; Bode phase plots for **(d)** DPPC, **(e)** DPPC/AuNP- HfUS liposomal systems and **(f)** DPPC/AuNP-LfT.

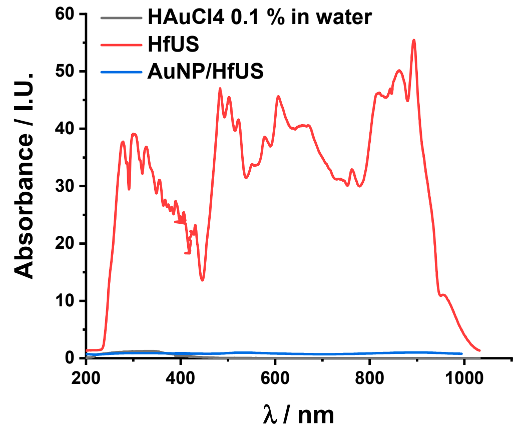

(a)

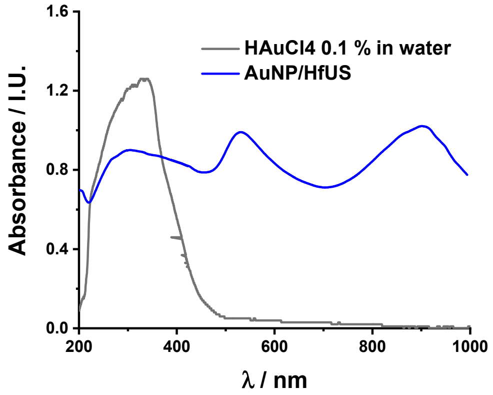

(b)

Figure S9. UV-Vis absorption spectra for: (**a**) diluted Hf US plant extract (4%), 0.01 % $HauCl_4$ aqueous solution and washed 0.01 % AuNP/HfUS colloidal solution. (b) magnification of (a) for 0.01 % $HauCl_4$ aqueous solution and washed 0.01 % AuNP/HfUS colloidal solution.

Table S1. Equivalent circuit parameter values obtained by fitting the impedance spectra from Figure 8 and Figure S6.

System	R_Ω / Ω	R_{ct} / kΩ	CPE_{dl} / µF $s^{\alpha-1}$	α
DPPC	11.28	454.4	0.179	0.899
DPPC + H_2O_2	11.41	267.8	0.283	0.885
DPPC/AuNP-HfUS	8.26	-	6.799	0.809
DPPC/AuNP-HfUS + H_2O_2	12.57	-	0.118	0.853
DPPC/AuNP-LfT	7.34	-	8.299	0.774
DPPC/AuNP-LfT + H_2O_2	10.25	-	0.272	0.836
